# Planetary health and non-communicable diseases—A converging global crisis

**DOI:** 10.3389/fpubh.2025.1674328

**Published:** 2026-01-12

**Authors:** Venkata Nagaraj Kakaraparthi, Paul Silvian Samuel, Shagun Agarwal, Kiran Velukuri, Nusarath Jaha Gurram Konda, Lalitha Kakaraparthi

**Affiliations:** 1 Department of Medical Rehabilitation Sciences, College of Applied Medical Sciences, King Khalid University, Abha, Saudi Arabia; 2 Department of Physiotherapy, Galgotias University, Greater Noida, India; 3 Department of Physiotherapy, The Apollo University, Chittoor, India

**Keywords:** environmental degradation, global health challenges, non-communicable diseases, planetary health, pollution, public health policy

## 1 Introduction

Planetary health is a nascent concept that takes into full consideration the interdependence between the health of human populations and the state of Earth's natural systems (1). In recent decades, the rapid degradation of the environment—distinguished by climate change, deforestation, pollution, and loss of biodiversity—has begun to exert profound impacts on human health. In addition, non-communicable diseases (NCDs), such as cardiovascular disease, diabetes, chronic respiratory diseases, and cancers, have become major contributors to mortality around the planet. Traditionally linked to lifestyles, there is increasing evidence that environmental degradation plays a critical, often overlooked, role in the epidemiology of NCDs (2).

Planetary health, first articulated by the 2015 Rockefeller Foundation–Lancet Commission, refers to “the health of human civilization and the state of natural systems on which it depends” (3). This framework highlights the interdependence between human wellbeing and Earth's ecological stability, emphasizing that disruptions to climate, biodiversity, land, oceans, and air quality directly translate into health risks (4). As these environmental systems degrade, exposure to climate stressors, pollution, food insecurity, and ecological imbalance amplifies vulnerabilities to a wide range of NCDs (5). The foundational study of the Commission and subsequent contributions from the Planetary Health Alliance provide the conceptual basis for understanding these interlinked human–environment relationships.

The concept of planetary health makes humankind fully aware that development or progress depends on a stable and functional environment. Environmental alterations from unsustainable activity, with the combustion of fossil fuel, industrial agriculture, and deforestation have worsened air quality, water security, food security, and overall ecosystem resilience. These modifications relate not only to biodiversity and climate stability but also to conditions constituting major risk factors for NCDs (6). For instance, cardiovascular and respiratory diseases are increasingly related to air pollution, while changes in the way food is produced and distributed add to altered eating patterns that further enhance the burden of obesity and diabetes (7).

Another such key trend that influences planetary health is urbanization, which contributes to sedentary living and increased stress levels, which in turn favor the development of NCDs. Indeed, the overlap between planetary health and NCDs is even more concerning because low- and middle-income countries carry a disproportionately greater share of this burden. This occurs because these countries often experience higher exposure to environmental risks—such as air pollution, unsafe water, poor waste management, and climate-related shocks—while simultaneously having weaker health systems unable to prevent, diagnose, or manage chronic diseases effectively. In addition, rapid urbanization, reliance on biomass cooking fuels, limited green spaces, and inadequate regulatory frameworks increase population-level vulnerability to NCDs. These environmental stressors interact with social and economic disadvantages, making individuals in LMICs more susceptible to developing severe and early-onset NCDs. Many of these countries do not have the full capacity or capability to reduce harm to the environment and provide optimal care for those with non-communicable diseases (8).

Along with these, animal agriculture contributes substantially to climate change, accounting for a large share of global methane emissions, extensive land use, deforestation, and high-water consumption. Ruminant livestock such as cattle produce methane during digestion, while feed production and manure management further amplify emissions. These environmental impacts make dietary choices a key lever in mitigating climate change.

This paper will consider the critical intersection of planetary health and NCDs, focusing on how changes in the environment influence the patterns of diseases and health outcomes. Mapping the environmental determinants of NCDs and discussing novel, sustainable approaches will make the discussion a comprehensive approach to health for both people and the planet. Planetary health is no longer strictly an ecological or scientific challenge but a public health imperative, and must be met with collaborative and cross-sectoral solutions to ensure a healthier, more sustainable future for all.

## 2 Planetary health influences on NCDs

### 2.1 Air pollution and respiratory diseases

Air pollution is a significant public health issue with major effects on respiratory health. Air is a complex mixture of solid particles, liquid droplets, and gases, with major air pollutants, including particulate matter (PM2.5 and PM10), nitrogen dioxide (NO_2_), sulfur dioxide (SO_2_), carbon monoxide (CO), and ozone (O3) at ground level. Outdoor and indoor exposure to these pollutants has been linked to respiratory diseases such as asthma, chronic obstructive pulmonary disease (COPD), lung cancer, and respiratory infections (9).

Recent empirical studies demonstrate strong associations between air pollution and NCD outcomes. Kim et al. reported significant links between fine particulate matter (PM2.5) exposure and neurological disorders, including cognitive decline and neuroinflammation (10). Similarly, Kumar et al. highlighted the role of emerging pollutants in increasing the incidence of cardiac arrhythmias and ischemic events (11). These findings underscore how environmental degradation directly shapes chronic disease pathways.

#### 2.1.1 Particulate matter (PM2.5 and PM10)

**Figure 1:**
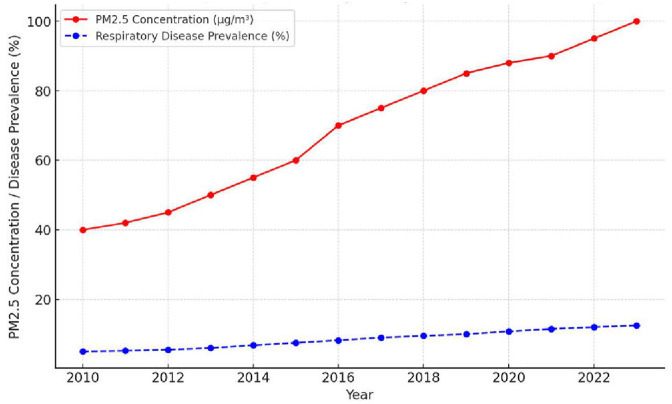
Trend of air pollution (PM2.5) and respiratory disease prevalence (2010–2023).

The main sources are from combustion engines (vehicles), industrial processes, wildfires, and household solid fuel use. The small size of PM2.5 enables it to reach deep into the lungs, reaching the alveoli and even possibly entering the bloodstream. Chronic exposure can provoke inflammation, reduce lung function, and worsen respiratory illnesses such as asthma, bronchitis, and COPD. It is linked to diseases such as asthma, COPD, lung cancer, and acute lower respiratory infections (Figure 1) (12).

#### 2.1.2 Nitrogen dioxide (NO_2_)

Mainly released from traffic, factories, and power plants. Short-term exposure to *NO*_2_ can irritate the airways, while long-term exposure can increase the risk of respiratory infections, wheezing, reduced lung function, and the development of asthma in children (13).

#### 2.1.3 Ozone (O3)

Created when pollutants from vehicles, power plants, and industry react with sunlight. Ozone is a strong irritant to the lungs. In high amounts, particularly in the summer, ozone can lead to coughing, irritation of the throat, and decreased lung function. It also increases the risk of attacks in asthmatic children and inflammation of lung tissue, as well as decreases lung function in children and older adults (14).

#### 2.1.4 Sulfur dioxide (SO_2_)

It is emitted from the burning of fossil fuels (coal, oil) in power plants and other industrial processes. SO_2_ irritates the respiratory system, causing bronchospasm and constriction of the airways. This is especially harmful for individuals with pre-existing respiratory conditions. It was also linked to asthma, wheezing, and an increased risk of respiratory tract infections.

#### 2.1.5 Carbon monoxide (CO)

Incomplete combustion of carbon-containing fuels (vehicle emissions, biomass burning, and indoor heating systems) is the main source of carbon monoxide. It binds to hemoglobin in the blood and prevents it from carrying oxygen, decreasing organ oxygen delivery. Long-term exposure may cause cognitive impairment and respiratory distress. It is associated with diseases such as respiratory distress, impaired oxygen transport, and increased mortality in high-risk groups (15).

#### 2.1.6 Vulnerable populations to air pollution

Children are particularly susceptible to air pollution because their lungs, airways, and immune defenses are still maturing, and they breathe more rapidly than adults. This combination results in a higher dose of pollutants per body weight and less physiological capacity to neutralize or clear these toxins. When developing lungs are exposed to pollutants, growth can be stunted with lasting consequences. The lungs' ability to filter and clear pollutants declines with age, so older adults are at greater risk of chronic obstructive pulmonary disease as well as lung infections (16). Air pollution exposure during pregnancy is linked to low birth weight, preterm birth, and respiratory problems in newborns (17).

#### 2.1.7 Income and geographic disparities

Low-income households are disproportionately exposed to environmental hazards because they frequently reside near industrial sites, occupy substandard housing, or depend on biomass fuels for cooking. Countries with weaker environmental regulations suffer more air pollution (18). Vehicle pollution is a big issue in urban spaces, whereas rural regions are plagued by indoor air pollution due to the burning of biomass (19).

#### 2.1.8 Global burden of disease

According to the World Health Organization (WHO), 7 million people lose their lives due to exposure to air pollution every year (20). Air pollution is responsible for 29% of lung cancer deaths, 24% of deaths from stroke, 25% of deaths from heart disease, and 43% of deaths from COPD (21). The disease burden from air pollution is highest in India and China, which have the highest urbanization (22).

These trends in air pollution (PM2.5) and respiratory disease prevalence (2010–2023) are illustrated in Figure 1 (23).

### 2.2 Climate change and food insecurity

Direct and indirect effects of climate change on the global food system are major threats to food security that pose a substantial risk to NCDs. Dry and wet weather, erratic rainfall patterns, and extreme weather events such as droughts and floods can impact agricultural production, resulting in lower crop yields and food shortages (24). This results in communities having to rely on more affordable, energy-dense, and nutrient reactive foods, which substantially increases the risk of NCDs such as diabetes, obesity, and cardiovascular disease (25).

Food insecurity from climate change has significant health consequences, including undernutrition and lack of micronutrient availability due to degrading soil fertility, increasing atmospheric CO_2_, which dilutes the nutrient density of crops, and exposing plants to heat and water stress. This leads to lower levels of essential nutrients such as iron, zinc, folate, and vitamin A in commonly consumed staple foods (26). Lower crop yields reduce the supply of fresh fruits and vegetables and necessary staple foods, resulting in malnutrition (27). Along with this, nutritional inadequacies build up over time and lend themselves to an increased burden of NCDs, as the health issues from poor nutrition among children manifest across their lifetime (28).

#### 2.2.1 Heat exposure and health outcomes

Evidence from large-scale epidemiological analyses supports these associations. Chersich et al., in a systematic review and meta-analysis, found that high temperature exposure during pregnancy significantly increases the risk of preterm birth, stillbirth, and low birth weight (29). Such findings highlight the growing vulnerability of maternal and neonatal populations to climate-related heat stress.

#### 2.2.2 Urbanization and sedentary lifestyles

The endemic sedentary lifestyle in many regions of the world and the urbanization process of many cities contributed to the alarming rise in overweight, obesity, cardiovascular diseases, and diabetes (30). Residents living in these environments have fewer safe or accessible spaces for walking, jogging, or outdoor exercise, which reduces their overall physical activity and contributes to higher levels of sedentary behavior (31). Sedentary lifestyles are compounded by the proliferation of desk jobs, long hours of sitting in traffic, and over-dependence on cars. This change of lifestyle is linked to an increase in obesity and metabolic diseases (32).

Cardiometabolic vulnerability is also supported by emerging evidence. Bai et al. reported that long-term exposure to PM2.5 and nitrogen dioxide significantly increases the risk of hypertension, atherosclerosis, and metabolic syndrome, with clear dose-dependent associations. These mechanistic pathways reinforce the need to address environmental exposures as part of chronic disease prevention strategies (33).

In addition to physical health issues, urbanization impacts mental health, leading to more cases of depression, anxiety, and other mental health disorders that fall under mental health. Life in urban areas experiencing congestion and pressure influences mental health because of disconnection from nature (34). Low-income communities in densely populated metro regions are often subjected to never-ending noise, reduced air quality, over-stressed public spaces, and lack of green space that exacerbates that stress. Having cities with green spaces, pedestrian-friendly infrastructure, and community-engagement systems are solutions that take care of both physical and mental health from an urban planning perspective.

### 2.3 Water contamination and food safety

Water contamination poses significant risks to food safety and public health, particularly through industrial discharge, agricultural runoff, and plastic pollution. These sources introduce harmful substances such as heavy metals, pesticides, and microplastics into water systems, which can infiltrate agricultural produce and the food chain (35). Heavy metals such as arsenic, lead, and mercury, often found in contaminated water, accumulate in crops irrigated with such water or fish raised in polluted rivers and seas (36). For instance, arsenic contamination has been strongly linked to skin cancer and cardiovascular diseases, while lead exposure can impair cognitive development, especially in children, and contribute to hypertension and other metabolic disorders in adults. This requires stricter regulations on industrial and agricultural waste, investments in water purification technologies, and awareness campaigns to promote safe water practices in vulnerable regions.

### 2.4 Biodiversity loss and emerging zoonotic diseases

The consequent loss of biodiversity from habitat destruction, deforestation, and human encroachment on natural ecosystems has consequences for public health. Among the biggest challenges are zoonotic diseases, diseases that are transmitted from animals to humans (37). The destruction of ecosystems overturns the balance of wildlife and human numbers, providing opportunities for pathogens to jump species. Events such as the COVID-19 pandemic illustrate the power of these diseases to spread rapidly and destabilize nations.

In this regard, they have indirect but important implications for non-communicable disease (NCD) control. During pandemics, mass health care systems become overwhelmed. Many chronic disease treatments and interventions suddenly discontinue. During outbreaks of infectious diseases, preventive care, routine visits, and non-communicable disease (NCD) management are among the priorities to be deprioritized as staff and resources are mobilized for emergency measures (38).

These empirical observations align with global climate–health assessments, including the Lancet Countdown 2025 report, which documents rising climate-linked risks for cardiovascular, respiratory, and metabolic diseases across multiple regions (39).

### 2.5 Policy implications and solutions

#### 2.5.1 Health system interventions

In this context, integration refers to embedding climate-risk measures, such as heat alerts, air-quality monitoring, and emergency readiness, within routine NCD prevention and care to create a proactive, resilient health system (40). Public health infrastructure must be designed to keep essential services functioning during climate crises, allowing health systems to manage both acute climate-related illnesses and ongoing NCD care without disruption (41). Improved accessibility of health services and insurance coverage, which would benefit many vulnerable people, ensures equitable delivery, as this will bridge the gaps created by climate impacts.

#### 2.5.2 Environmental interventions

Effective environmental policies form the bedrock to mitigate the health impacts of climate change. A shift toward renewable energy use, coupled with effective implementation of pollution control, drastically reduces air quality-related burdens on human health, reducing further diseases associated with respiratory complications (42). Planning smart and walkable cities within green spaces can help people to support people in becoming more active, boosting both physical health and mental health effectively.

#### 2.5.3 Behavioral interventions

Shifting society's behavior will be key to building resilience that would help avert both the challenges arising from climate change and the NCDs (43). Facilitating healthy eating patterns, such as the consumption of sustainable, plant-based diets, reduces agricultural emissions and results in a reduction in obesity and diseases of the metabolic type, while an active lifestyle-boosting infrastructure in built-up areas results in increasing public participation in physical activity (44). There is a great need for psychosocial support services, especially for people under climate change-induced stress and displacement, to help those impacted cope with their compound burden regarding mental health and physical health issues.

#### 2.5.4 Role of global institutions

Global institutions should be the focal point for aligning health goals with climate goals. Such organizations as the WHO, UNDP, and the World Bank should be at the forefront of building planetary health principles into the framework of NCD prevention (45). International cooperation should be promoted to address this critical issue for addressing the interconnected crises of climate change and public health, and ensuring a healthier and more sustainable future for all (46).

## 3 Conclusion

The simultaneous rise of planetary health challenges and NCDs represents a critical global concern that requires rapid, coordinated, and strategic action. In other words, human activity has compounded this natural breakdown of the central structures supporting life on the planet, thus leading to a rise in the NCD burdens. This is a crisis that can only be tackled with a whole-of-society approach, as air and water pollution, climate change, urbanization, and loss of biodiversity increasingly determine the distribution of disease. Such measures would ensure that policy approaches to the adoption of cleaner energies, efficient use of food, active transport, and minimal generation of waste, designing further policies, and together contribute to the declines in drivers of NCDs while protecting the environment. Moreover, full health equity recognition will be required and support for low- and middle-income countries as part of joint global progress. Therefore, it is time to act; planetary health needs to be a core feature of public health approaches to combat the global burden of NCDs and the planet's future.
